# Secretome improves testosterone and androgen-binding protein production in testicular dysfunction rats induced by cisplatin

**DOI:** 10.5455/javar.2021.h561

**Published:** 2021-12-13

**Authors:** Dwi Sunu Datrianto, Teguh Budipitojo, Surya Agus Prihatno

**Affiliations:** 1 Faculty of Veterinary Medicine, Universitas Gadjah Mada, Yogyakarta, Indonesia; 2 Department of Anatomy, Faculty of Veterinary Medicine, Universitas Gadjah Mada, Yogyakarta, Indonesia; 3 Department of Reproduction and Obstetric, Faculty of Veterinary Medicine, Universitas Gadjah Mada, Indonesia

**Keywords:** ABP, cell, regeneration, testes

## Abstract

**Objective:**

This study was determined to see the effects of secretome on cellular production of testosterone and androgen-binding protein (ABP) using immunohistochemistry in testicle dysfunction due to cisplatin.

**Materials and Methods:**

Forty-eight rats were divided into four groups: the healthy group, the group with testicular dysfunction, the secretome-treated group with 0.2 ml/kg body weight (BW), and the secretome-treated group with 0.3 ml/kg BW. The immunohistochemistry staining method is used to find out testosterone and ABP reactivity in tissue organs.

**Results:**

Very strong testosterone and ABP immunoreactivity were found in Leydig cells of normal testes. While in the Leydig cells of cisplatin-induced testicles, testosterone and ABP immunoreactivity were not observed. Testosterone and ABP were observed 1 week after the second secretome injection. The number of testosterone-immunoreactive cells in the low dose group from week 1 to 3 was 0, 19, and 32, respectively. From week 1 until week 4, the high dose group was 0, 29, 33, and 65, respectively. The number of ABP-immunoreactive cells from the first week until the third week in the low dose group was 0, 28, and 34, respectively. The high dose group from the first week until the fourth week was 0, 26, 58, and 83, respectively. The number of cells that produce testosterone and ABP increased from week 2 to 4.

**Conclusion:**

The administration of secretome could increase the number of immunoreactive cells toward testosterone and ABP in testicular dysfunction.

## Introduction

Testicular dysfunction can affect fertility and result in an insufficient supply of androgen (testosterone). Numerous environmental and internal factors have an effect on testicular function; one of the most significant is exposure to potentially dangerous drugs such as cisplatin [1]. Cisplatin is a chemotherapeutic agent used to treat ovarian cancer, cervical cancer, malignancies of the urinary veins, and testicular tumors. Cisplatin, on the other hand, has some adverse effects. Cisplatin injection at a dose of 3 mg/kg body weight (BW) decreased testicle size, semen volume, sperm count in the epididymis, and motile sperm percentage in one study. This adverse effect has been linked to a decrease in testicular function [2]. As a result, cisplatin injection could result in testicular damage [3]. Some researchers used cisplatin’s unfavorable effect to create an animal model of testicular failure in order to discover new therapies for this male reproductive problem.

Secretome is a potential regenerative agent that has recently attracted the attention of a huge number of researchers. Secretome injection hastened wound closure and increased fibroblasts, collagen fibers, and blood vessels in the treated group compared to the control group [4]. In another study, secretome injection enhanced the motility of spermatozoa and the regeneration of spermatogenic cells in rats with testicular dysfunction [5]. Additionally, secretome has been studied as a potential therapeutic paradigm for central nervous system neurodegenerative diseases [6]. Secretome infusion appears to be able to modulate certain cytokines such as IL6 and IL10 during the testicular regeneration process [7]. It is not limited to soft organs; it has been demonstrated that secretome therapy promotes osteogenic differentiation in animals with faulty osteogenesis [8].

Numerous clinical trials have been carried out to demonstrate the secretome’s usefulness in the treatment of a variety of degenerative diseases. Additionally, our team has shown regeneration of spermatogenesis in mice with testicular failure. However, the testosterone and androgen-binding protein (ABP) production processes required for spermatogenesis have never been verified. This work demonstrated testosterone and ABP synthesis in rats with cisplatin-induced testicular impairment following secretome injection.

## Materials and Methods

### Ethical approval

The Ethical Committee of Universitas Gadjah Mada approved this study with reference number 00035/04/LPPT/V/2017.

### Experimental animal

To acclimatize mice, 7 days of basic meals and *ad libitum* water consumption were used. Cisplatin was given in a volume according to the weight of the rat. To induce testicular dysfunction, cisplatin was injected intraperitoneally three times every 3 days. Rats were randomly allocated to one of four groups: healthy, testicular dysfunction, testicular dysfunction treated with secretome at a 0.2 ml/kg BW dose, or testicular dysfunction treated with secretome at a 0.5 ml/kg BW dose. Cisplatin was administered intraperitoneally three times at a dose of 3 mg/kg BW with a 3-day interval between each dose to induce malfunction. Secretome was injected four times into rats in the secretome-treated groups at 7-day intervals. Rats were slaughtered every week after secretome was injected with ketamine (75 mg/kg BW).

One week after secretome injection, testes tissue samples were collected each week. To determine testosterone and ABP distribution in the testes, testes tissues were fixed in Bouin’s solution for 24 h, processed using the paraffin method, and stained using the immunohistochemical method. A light microscope was used to examine immunohistochemical preparations, and the results were assessed descriptively.

### Testosterone and ABP immunohistochemical staining

Testicular testosterone and ABP immunolocalizations were seen using a streptavidin–biotin complex detection kit (Starr Trek Universal HRP Detection System Kit, Biocare Medical®, USA). The tissue samples were deparaffinized, dehydrated, and rinsed for 10 min in running water. At room temperature, the tissue sample was incubated in a blocking endogenous peroxidase solution for 20 min and then washed three times with phosphate buffer saline (PBS) for 5 min each. Following that, the tissue slides were incubated for 20 min at room temperature in Biocare’s background sniper solution. The tissue slides were incubated overnight at 4°C with testosterone 19, polyclonal antibody (1:10) from Mybiosource®, San Diego, CA, and ABP/sex hormone binding globulin polyclonal antibody (1:500) from BIOSS®, Massachusetts, USA. One slide was kept as a negative control where no antibodies were given.

The second day begins with the slides being removed from the refrigerator and left at room temperature for 10 min before being soaked in PBS three times for 5 min each. The antibody was then incubated with one drop of Trekkie Universal Link antibody and covered with parafilm for 25 min. Additionally, the slides were soaked in PBS three times for 5 min each. For 25 min, a drop of Trek Avidin horseradish peroxidase (HRP) was given and covered with parafilm. Three times for a total of 5 min, slides were soaked in PBS. To visualize the reactive component of the tissue, slides were dropped with 30 μl of 3,3’-diaminobenzidine substrate and observed under a microscope. For 1 min, counterstain was carried out with hematoxylin. Then, they were rinsed for 10 min under running water and the object was mounted.

### Data analysis

The testosterone and ABP histochemistry staining findings were examined semi-quantitatively to determine the color intensity distribution. Four levels of color intensity are defined as follows: extremely strong, strong, medium, and weak. Establishing intensity standards requires comparing all photographs and then categorizing them according to their color intensity. The staining results were viewed under a 40× magnification light microscope. Each week, the number of testosterone- and ABP-reacting cells was determined and examined. Manual counting of immunoreactive cells was carried out using a light microscope. Calculation of five field views at a magnification of 40× for each sample was carried out.

## Results and Discussion

### Testosterone immunoreactivity

Testosterone immunoreactivity was extremely strong in normal testes, particularly in Leydig cells situated between seminiferous tubules. Negative controls lacked immuno-reactivity (brown) to testosterone. There was a shift in the intensity of testosterone immunoreactivity following secretome induction; testosterone reactivity increased and remained stable 1 week after the second secretome injection, at both high and low doses.

**Figure 1.**
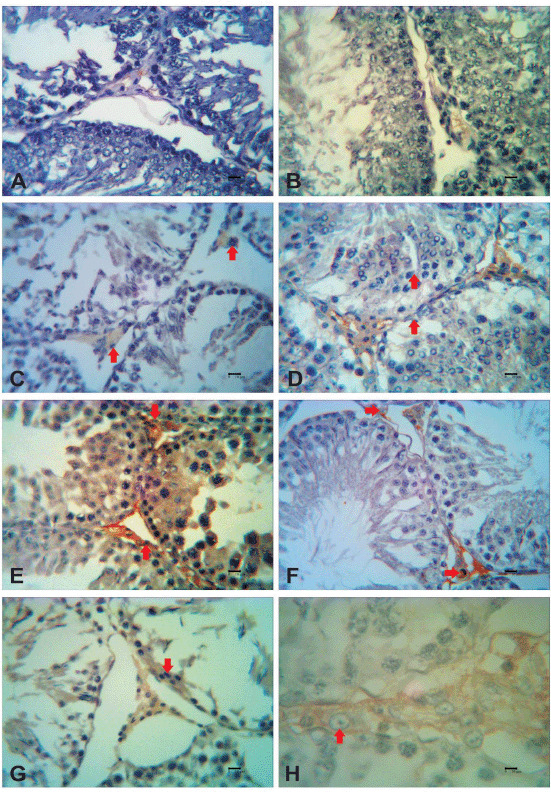
Testosterone immunoreactivity after secretome injection in testicular dysfunction rats treated with secretome. The reactivity of testosterone was detected in the interstitial cells of the testis (red arrows). Immunoreactivity of testosterone was not detected 1 week after the first secretome injection in the low dosage group (A) and high dosage group (B). One week after the second secretome injection, in the low dosage group (C), the immunoreactivity of testosterone was moderate; meanwhile, in the high dosage group (D), the immunoreactivity of testosterone was strong. One week after the third secretome injection, in the low dosage (E) and high dosage group (F), the immunoreactivity of testosterone was strong. One week after the fourth secretome injection, in the high dosage group (G), the immunoreactivity of testosterone was strong. There was strong reactivity of testosterone in Leydig cells with 40× magnification (H).

One week after the initial secretome injection, testosterone immunoreactivity remained unchanged. However, 1 week after the second induction of the secretome, testosterone immunoreactivity increased in both low and high doses. After the third and fourth secretome injections, the immunoreactivity persisted for up to 1 week. Unfortunately, testicular samples taken during the fourth secretome induction at low levels could not be studied. A semi-quantitative analysis of testosterone immunoreactivity was carried out. The immunohistochemical labeling of testosterone in each group of mice is shown in Figure 1.

**Figure 2.**
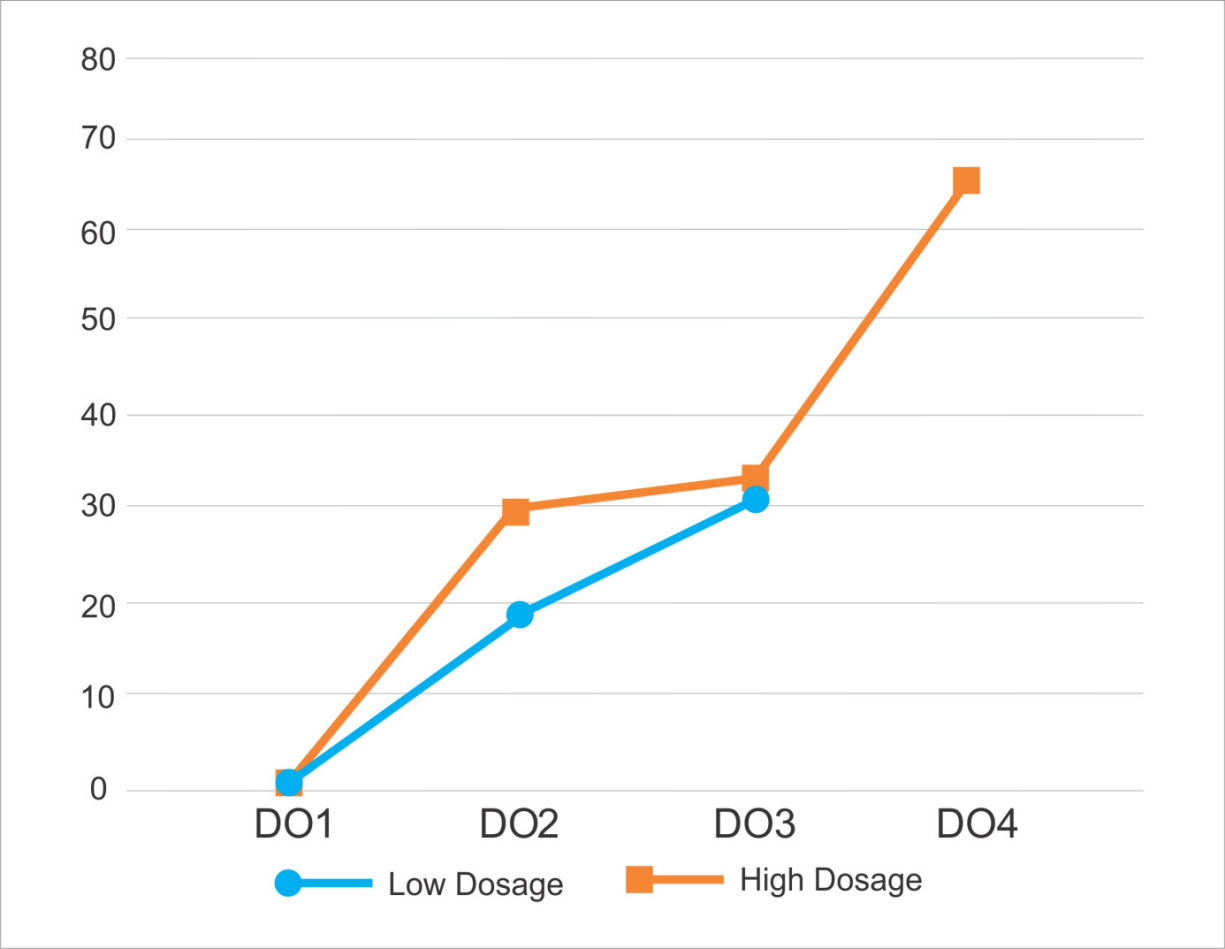
The results of calculating testosterone immunoreactive cells in testicular dysfunction rats treated with secretome. There was an increase in the number of cells that were immunoreactive with testosterone starting from 1 week after the second secretome injection (DO2), third (DO3), and fourth (DO4) in the 0.5 ml/kg BW (■) dosage group. There was an increase in the number of cells that were immunoreactive with testosterone starting from 1 week after the second secretome injection (DO2) and third (DO3) in the 0.2 ml kg^−1^ (•) dosage group. One week after the first secretome injection (DO1), the immunoreactive cells of testosterone were not observed.

Quantitative analysis revealed an increase in the number of cells in the secretome-treated group. Along with the intensity variations, the number of testosterone immunoreactive cells increased. In the first to third low dosage secretome inductions, the number of immunoreactive testosterone cells was 0, 19, and 32 in five fields of view at 40× magnification. Whereas the number of immunoreactive testosterone cells at the induction of the first to fourth high dose secretome was 0, 29, 33, and 65 in the five fields of view at 40× magnification, the number of immunoreactive testosterone cells at the induction of the first to fourth high dose secretome was 0, 29, 33, and 65, respectively. In the second week, there was a significant difference between low and high doses (asymp. sig. 0.046 (*p* < *α*), but in the third week, the data were normally distributed (asymp. sig. 0.63 (*p* > 0). The findings of the testosterone immunoreactive cell count are shown in Figure 2.

### ABP immunoreactivity

ABP exhibited a significant immunoreactivity in normal testes, as indicated by brown staining of the Leydig cells. There was no brown coloration in the negative control, indicating immunoreactivity. There was a shift in the strength of ABP immunoreactivity following secretome induction; ABP reactivity increased 1 week after the second secretome injection, at both high and low doses.

**Figure 3.**
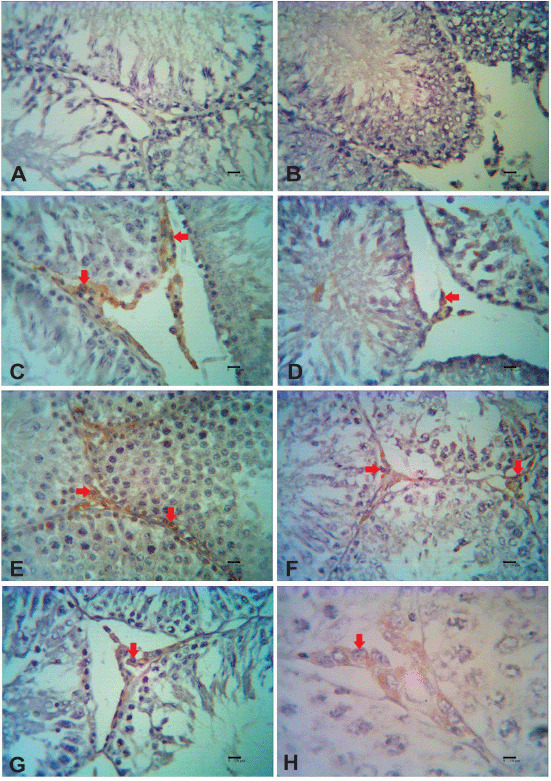
ABP immunoreactivity after secretome injection in testicular dysfunction rats treated with secretome. Reactivity of ABP was detected in the interstitial cell of the testis (red arrows). Immunoreactivity of the ABP was not detected for 1 week after the first secretome injection in the low dosage group (A) and high dosage group (B). One week after the second secretome injection, in the low dosage group (C), the immunoreactivity of ABP was strong; meanwhile, in the high dosage group (D), the immunoreactivity of ABP was moderate. One week after the third secretome injection, in the low dosage (E) and high dosage group (F), the immunoreactivity of ABP was strong. One week after the fourth secretome injection, in the high dosage group (G), the immunoreactivity of ABP was strong. There was strong reactivity of ABP in Leydig cells with 40× magnification (H).

One week after the initial secretome injection, no change in ABP immunoreactivity was observed. However, 1 week following the second secretome induction, the strength of ABP immunoreactivity increased significantly in both low (strong immunoreactivity) and high doses (moderate immunoreactivity). After the third and fourth secretome injections, the immunoreactivity persisted for up to 1 week. Unfortunately, testicular samples taken during the fourth secretome induction at low levels could not be studied. The result of ABP immunohistochemistry staining in each group of mice can be seen in Figure 3.

**Figure 4.**
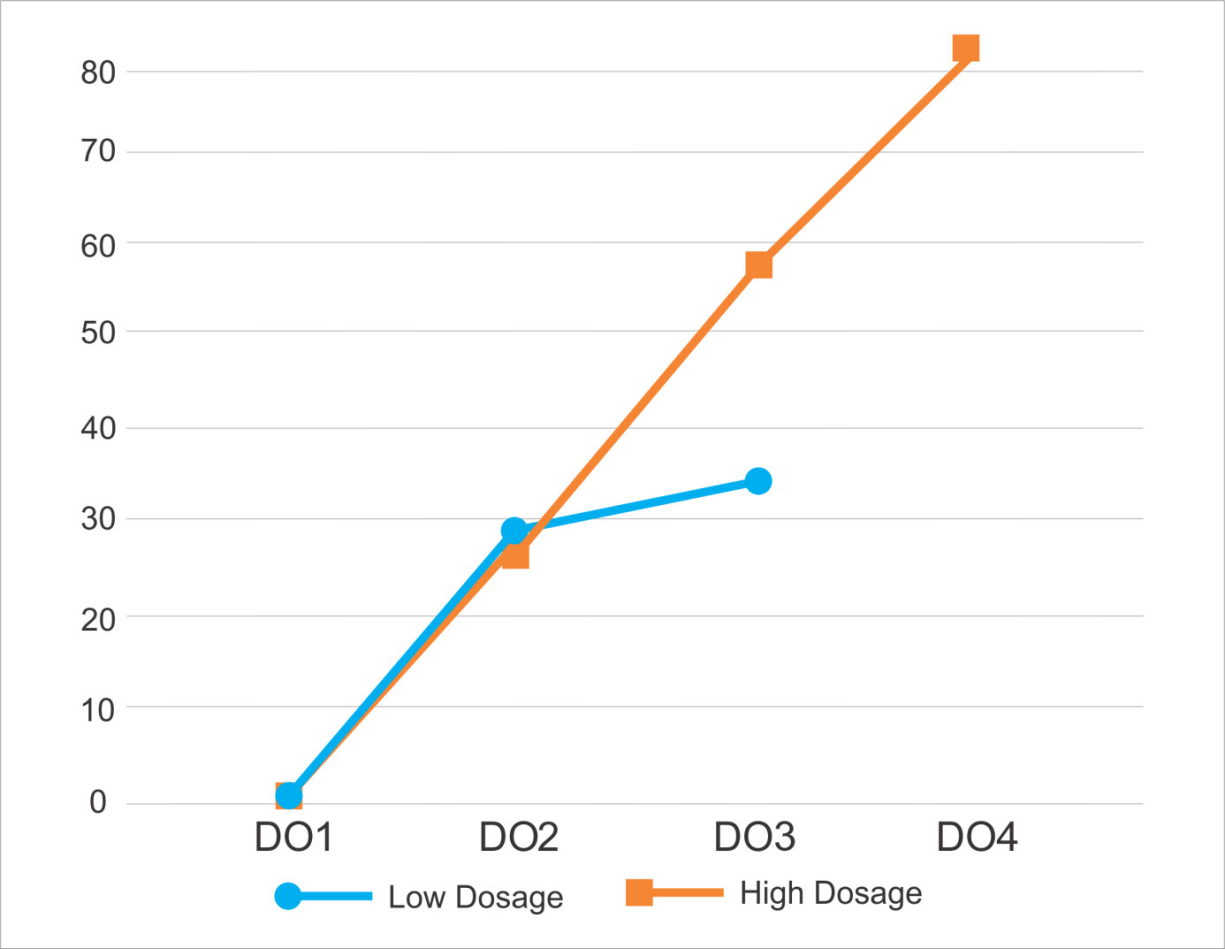
The results of calculating immunoreactive ABP cells in testicular dysfunction rats treated with secretome. There was an increase in the number of cells that were immunoreactive with ABP starting from 1 week after the second secretome injection (DO2), third (DO3), and fourth (DO4) in the 0.5 ml kg^−1^ (■) dosage group. There was an increase in the number of cells that were immunoreactive with ABP starting from 1 week after the second secretome injection (DO2) and third (DO3) in the 0.2 ml/kg BW(•) dosage group. One week after the first secretome injection (DO1), the immunoreactive cells of ABP were not observed.

Quantitative analysis revealed an increase in the number of cells in the secretome-treated group. Along with the intensity variations, the number of ABP immunoreactive cells increased. In the first to third low dose secretome inductions, the number of ABP immunoreactive cells was 0, 28, and 34 in five fields of view at 40× magnification. Whereas in the five fields of view at a magnification of 40×, the number of ABP immunoreactive cells was 0, 26, 58, and 83 at the time of induction of the first to fourth high dose secretome, respectively. In the second week, the difference between low and high dosage was normally distributed with asymp. sig. 0.197 (*p* > 0), but in the third week, the difference between low and high dosage was significantly different with asymp. sig. 0.046 (*p* < *α*). Figure 4 shows the results of the cell count that was immunoreactive to ABP.

No cells were immunoreactive against testosterone or ABP in the group with testicular dysfunction. Cisplatin-induced testicular dysfunction results in the death of germ cells, shrinkage of the testicles, Leydig cell dysfunction, infertility, decreased libido, and decreased testosterone production [9,10]. Cisplatin-induced testicular dysfunction arises as a result of cisplatin-induced oxidative stress and apoptosis in animal and human cells. It will increase the amount of reactive oxygen species (ROS) in the tissues. Increased ROS levels in tissues result in increased oxidative stress, leading cells to oxidize and finally undergvo apoptosis. When these cells die, ABP and testosterone are no longer generated. Another study reported that cisplatin administration dramatically lowered the serum testosterone level in testicular dysfunction rats. Cisplatin is toxic to Sertoli cells; as a result, ABP synthesis is decreased as a result of Sertoli cell damage caused by cisplatin injection [8].

One week following the second secretome injection, tissue repair occurred as indicated by the intensity of the brown color of the Leydig cells found in the interstitial part of the testis, meaning that an androgen hormone synthesis process occurred. Secretome administration has not been shown to have chemoattractive, anti-apoptotic, anti-tumorigenic, anti-fibrotic, anti-inflammatory, pro-angiogenic, neuroprotective, or antibacterial properties [11,12]. The improvement of dysfunctional testicular tissue treated with secretome occurred because the secretome contained the vascular endothelial growth factor, which was involved in the testicular process of angiogenesis. Another growth factor was the platelet-derived growth factor, which stimulated Leydig cell proliferation in the testes, resulting in testosterone production. Additionally, this hormone activated the 17αHSD enzyme, which catalyzed the conversion of dehydroepiandrosterone to androstenediol throughout the steroidogenesis process. Transforming growth factor beta (TGFα) was present in the secretome, which aided Leydig cells to perform efficiently. The secretome contained epidermal growth factor, which stimulated the 3αHSD enzyme, which converted androstenediol to testosterone [13].

Quantitative investigation revealed an increase in the number of testosterone-reactive cells in both secretome dosage groups. This is because the secretome regenerates spermatogenic cells in the injured seminiferous tubules caused by cisplatin injection, allowing the hormone-producing and spermatogenesis processes to resume. *In vitro* experiments have shown that these cells secreted growth factors into polymorphonuclear cell cultures, establishing it as a possible repair agent via paracrine signals and secretome as a protein secreted by cells, organs, or organisms that can respond to heal injured tissue [6,14,15]. Due to their ability to move to injured tissue and decrease the inflammatory response, mesenchymal stem cells (MSCs) are an effective agent for tissue healing [16]. Wound healing and tissue restoration occur as a result of the paracrine activity of secretome’s bioactive factors [17]. Additionally, the secretome inhibits cell death by lowering pro-apoptotic factor activity (Bax, caspase-3) while enhancing anti-apoptotic factor activity (BCL-2) and improving the local microenvironment in injured tissues [18].

Tissue repair occurred 1 week after the second secretome injection; the intensity of the brown color of the Leydig cells suggested the presence of an ABP production process. Not only did the color intensity change, but there was also an increase in the number of reactive cells to ABP in both secretome dosage groups, as shown by quantitative analysis. Sertoli cells generated ABP and were found in the seminiferous tubules; immunohistochemical labeling revealed that cells immunoreactive with ABP were Leydig cells, as ABP bound testosterone with high affinity and specificity and sustained high intratesticular testosterone concentrations. Interstitial testosterone is the amount of testosterone linked with Leydig cells that are bound to ABP.

The results indicated that testicular tissue had improved, as evidenced by the presence of cells immunoreactive to ABP. Spermatogenesis occurs as a result of a complicated interaction between spermatogenic cells and testicular interstitial cells, which is facilitated by an appropriate environment. These include endocrine, paracrine, and autocrine systems [7]. The secretome contains basic fibroblast growth factor, a growth factor that promotes Sertoli cell proliferation in the testes and also stimulates Sertoli cells to make glutatonin. Glutatonin was an anti-oxidant for cells that reduced the number of free radicals in the tissue, reducing oxidative stress and the adverse effect of cisplatin. Insulin growth factor was identified in the secretome, optimizing the anterior pituitary’s synthesis of follicle stimulating hormone, enhancing steroidogenesis stimulation.

TGFα was found in the secretome, and it played a role in enhancing Sertoli cell activity [19]. The secretome contained growth factors and interleukins that influenced intrapancreatic regeneration by activating and recruiting circulating stem cells and progenitor cells to injured sites, where they collaborate to repair damaged tissue. Additionally, previous research has demonstrated that MSCs circulate in injured locations and develop specifically inside tissue [20].

## Conclusion

The extracts of MSC growth media were able to regenerate testes that had suffered from cisplatin-induced dysfunction, as determined by testosterone and ABP immunolocalization in testicular tissue.

## List of abbreviations

ABP, Androgen-binding protein; BW, Body weight; MSC, Mesenchymal stem cell; IL, Interleukin; HRP, Horseradish peroxidase; PBS, Phosphate buffer saline; ROS, Reactive oxygen species; TGFα, Transforming growth factor beta; BCL-2, B-Cell Lymphoma-2.
